# Adapting a practical EPR dosimetry protocol to measure output factors in small fields with alanine

**DOI:** 10.1002/acm2.14191

**Published:** 2023-11-03

**Authors:** Sebastian Höfel, Pauline Liebig, Michael K. Fix, Malte Drescher, Felix Zwicker

**Affiliations:** ^1^ Department of Chemistry and Konstanz Research School Chemical Biology University of Konstanz Konstanz Germany; ^2^ Klinik und Praxis für Strahlentherapie am Klinikum Konstanz Konstanz Germany; ^3^ Division of Medical Radiation Physics and Department of Radiation Oncology, Inselspital Bern University Hospital and University of Bern Bern Switzerland; ^4^ Department of Radiation Oncology Heidelberg University Hospital Heidelberg Germany; ^5^ Clinical Cooperation Unit Molecular Radiation Oncology German Cancer Research Center (DKFZ) Heidelberg Germany

**Keywords:** alanine, density effect, EPR dosimetry, output factor, small field, volume effect

## Abstract

**Purpose:**

Modern radiotherapy techniques often deliver small radiation fields. In this work, a practical Electron Paramagnetic Resonance (EPR) dosimetry protocol is adapted and applied to measure output factors (OF) in small fields of a 6 MV radiotherapy system. Correction factors and uncertainties are presented and OFs are compared to the values obtained by following TRS‐483 using an ionization chamber (IC).

**Methods:**

Irradiations were performed at 10 cm depth inside a water phantom positioned at 90 cm source to surface distance with a 6 MV flattening filter free photon beam of a Halcyon radiotherapy system. OFs for different nominal field sizes (1 × 1, 2 × 2, 3 × 3, 4 × 4, normalized to 10 × 10 cm^2^) were determined with a PinPoint 3D (PTW 31022) IC following TRS‐483 as well as with alanine pellets with a diameter of 4 mm and a height of 2.4 mm. EPR readout was performed with a benchtop X‐band spectrometer. Correction factors due to volume averaging and due to positional uncertainties were derived from 2D film measurements.

**Results:**

OFs obtained from both dosimeter types agreed within 0.7% after applying corrections for the volume averaging effect. For the used alanine pellets, volume averaging correction factors of 1.030(2) for the 1 × 1 cm^2^ field and <1.002 for the larger field sizes were determined. The correction factor for positional uncertainties of 1 mm was in the order of 1.018 for the 1 × 1 cm^2^ field. Combined relative standard uncertainties *u_c_* for the OFs resulting from alanine measurements were estimated to be below 1.5% for all field sizes. For IC measurements, *u_c_* was estimated to be below 1.0%.

**Conclusions:**

A practical EPR dosimetry protocol is adaptable for precisely measuring OFs in small fields down to 1 × 1 cm^2^. It is recommended to consider the effect of positional uncertainties for field sizes <2 × 2 cm^2^.

## 1 INTRODUCTION

In radiation therapy, precise and accurate dosimetry is required in clinical routine for correct commissioning of radiotherapy equipment, beam output calibration and quality assurance of dose delivery. Modern treatment techniques such as stereotactic radiosurgery, intensity modulated radiotherapy (IMRT) or volumetric modulated arc therapy (VMAT) frequently apply small radiation field sizes. A radiation field is usually considered as small if at least one of the following conditions is fulfilled: (i) lateral charged particle equilibrium is not established in the field center, (ii) there is partial occlusion of the primary photon source by the beam collimating devices on the beam axis, (iii) the dimensions of the detector are similar or larger than the field size in the plane of measurement.^1^ Dosimetry in small fields is cumbersome and prone to errors, since the influence of the dosimeter design (i.e. shape, size, mass density and composition) on its response increases with decreasing field size.^1^, ^2^, ^3^, ^4^ Hence, dosimeter specific effects, especially volume averaging and density effects, need to be evaluated and appropriate corrections have to be applied for accurate dosimetry.^1^, ^2^, ^3^, ^4^, ^5^, ^6^ Commissioning of modern treatment planning systems (TPS) includes, inter alia, measured data for output factors (OFs) in small fields. These measurements are usually performed during the machine commissioning as well as within the context of quality assurance. Inaccurately measured OFs may propagate to incorrect dose calculation and faulty dose delivery to patients.^7^

In clinical routine, ionization chambers (IC) are frequently used for dosimetry and corresponding standard procedures are well established. Specific codes of practice for dosimetry in small fields have been published (e.g. IAEA TRS‐483^1^ and DIN 6809‐8^2^). Electron paramagnetic resonance (EPR) dosimetry using powder‐pressed pellets of L‐alanine (ALA) has favorable characteristics for application in radiotherapy and generally requires only few and small corrections.^8^, ^9^, ^10^, ^11^ At present, however, this method is not widespread among radiotherapy departments, presumably due to the dependence on external laboratories on the one hand, and on the other hand due to high investment costs and high efforts for local implementations of precise EPR dosimetry.^8^, ^11^ ALA dosimetry is particularly recommended in small radiation fields since ALA is considered highly water equivalent,^3^, ^12^ thus reducing the impact of the density effect.^3^, ^5^, ^6^, ^13^, ^14^ However, depending on the dosimeter size and the field size, volume averaging effects are relevant. ALA has been used as reference for dosimetry in small fields,^3^, ^12^ that is, correction factors for different detectors were determined based on a comparison to the ALA response. In these studies, corrections only due to volume averaging were applied for ALA. However, there are studies that have claimed a non‐negligible density effect of ALA that demands for additional correction factors in situations where charged particle equilibrium (CPE) is lost, especially in the dose build‐up region or in small fields.^14^

In contrast to IC measurements, ALA dosimetry is a passive offline method. Therefore, correct positioning of ALA dosimeters on the central axis (CAX) of the beam (as required for OF measurements) is more challenging and subject to higher uncertainties. It is expected that uncertainties related to dosimeter positioning are becoming more important in small fields, especially when the field size approaches the dosimeter size.

In our previous work, we presented a practical EPR dosimetry system^8^ using commercial ALA dosimeters with a diameter of 4 mm and a height of 2.4 mm and demonstrated its routine applicability in radiotherapy.^15^, ^16^ The aim of the present work was to investigate the volume averaging effect and the impact of positional uncertainties on the accuracy of the established EPR dosimetry system when measuring OFs in small fields ranging from 1 × 1 to 4 × 4 cm^2^. A clinical 6 MV linear accelerator (Halcyon) with pre‐configured beam model (BM) is used exemplarily. Correction factors and dose uncertainties are derived from measured dose distributions using radiochromic film and IC resulting in an adapted EPR dosimetry procedure for OF measurements in small fields. The OFs and associated uncertainties obtained with ALA are compared to the BM data and to IC measurements following the IAEA‐AAPM International Code of Practice, TRS‐483^1^. Based on these findings, the influence of positioning uncertainties on the resulting OFs and the necessity of including further corrections related to the density effect for ALA are discussed.

## 2 METHODS

### 2.1 General methods

All irradiations were performed with a Halcyon (Varian Medical Systems, Palo Alto, CA, USA) treatment system providing a 6 MV flattening filter free (FFF) photon beam. Measurements were conducted for square nominal field sizes of 1 × 1, 2 × 2, 3 × 3, 4 × 4 and 10 × 10 cm^2^ in 10 cm phantom depth to eliminate the contribution of contamination electrons.^1^ The field size was defined at the source‐to‐detector distance (SDD) of 100 cm. The source‐to‐surface distance (SSD) was set to 90 cm. This setup met the output factor specifications of the pre‐configured BM in the Eclipse TPS. For OF measurements, commercial ALA pellets from Aérial (Illkirch, France) and a PinPoint 3D 31022 (PTW, Freiburg, Germany) ionization chamber (in the following referred to as “IC”) were used. The dosimeters were irradiated on the CAX in a MP3‐T water tank phantom (PTW, Freiburg, Germany). Since optical SSD indicators or a light field are not provided by the closed gantry system of the Halcyon,^17^, ^18^, ^19^, ^20^ MV imaging was applied to verify the vertical setup of the water tank. Depth dose curves and lateral dose profiles were measured with the Tandem electrometer T10011, the TBA control unit T41013 and the Mephysto mc^2^ software package (PTW, Freiburg, Germany). For final leveling and alignment of the water tank inside the Halcyon's closed gantry, lateral dose profiles of a 4 × 4 cm^2^ field at different off‐axis positions (± 1 cm) and depths (5 and 10 cm) were measured. Tilts were corrected by manually adjusting three leveling screws provided by the MP3‐T phantom which were accessible from the rear end of the bore until all profile offsets were below 0.4 mm in relation to the zero position.

Irradiations of gafchromic EBT3 films (Ashland Advanced Materials, Bridgewater, NJ, USA) were conducted in a RW3 (PTW, Freiburg, Germany) solid water slab phantom (30 cm x 30 cm x 15 cm). Vertical positioning was verified by a 90° MV image prior to placing the film in the slab phantom.

The irradiation field size was defined as equivalent square small field size $S_{clin}\;$according to TRS‐483^1^ and it was verified to match the nominal square field side length *s* within 1%.

OFs are also termed $\Omega_{Q_{clin},Q_{ref}}^{f_{clin},f_{ref}}$ and are determined via Equation (1) according to TRS‐483^1^. This notation indicates that a specific output factor Ω relates the absorbed dose to water in a clinical non‐reference field $f_{clin}$ of quality $Q_{clin}$ to the absorbed dose to water in the reference field $f_{ref}$ of quality $Q_{ref}$.

(1) $$\Omega_{Q_{\mathrm{clin}},Q_{\mathrm{ref}}}^{f_{\mathrm{clin}},f_{\mathrm{ref}}}=\frac{M_{Q_{\mathrm{clin}}}^{f_{\mathrm{clin}}}}{M_{Q_{\mathrm{ref}}}^{f_{\mathrm{ref}}}}\;k_{Q_{\mathrm{clin}},Q_{\mathrm{ref}}}^{f_{\mathrm{clin}},f_{\mathrm{ref}}}$$

$M_{Q_{clin}}^{f_{clin}}$ and $M_{Q_{ref}}^{f_{ref}}$ are the detector readings corrected for influence quantities for the clinical and reference field, respectively. In the present work, a nominal 10 × 10 cm^2^ square field was chosen as reference field. $k_{Q_{clin},Q_{ref}}^{f_{clin},f_{ref}}$ is the output correction factor comprising several sub‐factors that account for different effects such as volume averaging, fluence perturbation and spectral dependence of photon energy absorption.^3^ For ALA detectors, volume averaging is the dominant effect.^3^ In the current work, a volume averaging correction factor $k_{vol}\left(n\times n\right)$ was determined (cf. Section 3) for each field size (n×n). The effect of positional uncertainties was introduced as an additional correction factor $k_{pos}\left(n\times n\right)$ (cf. Section 4). For ALA, $k_{Q_{clin},Q_{ref}}^{f_{clin},f_{ref}}$ was assumed to be $k_{vol}\;$or the product $(k_{vol}\cdot$ $k_{pos})$. The disregard of possible density effects is discussed.

OFs were also determined from IC measurements via Equation (1) by applying published $k_{Q_{clin},Q_{ref}}^{f_{clin},f_{ref}}$ values.

Uncertainties presented in this work are of Type B according to GUM:1995.^21^ Combined uncertainties were estimated assuming that the different uncertainty components are uncorrelated.

### 2.2 Detector specific methods

#### 2.2.1 Ionization chamber

The used PinPoint 3D 31022 is an air‐vented IC suitable for small field dosimetry.^2^ It's sensitive volume of 0.016 cm^3^ has a radius of 1.45 mm and a length of 2.9 mm. IC measurements were conducted with the chamber axis in parallel with the beam direction for two reasons: (i) easy exchange of IC versus ALA detectors using the same detector holder in the same lateral position, (ii) availability of output correction factors. Field output correction factors for the PinPoint 3D 31022 were determined by Poppinga et al.^22^ for parallel chamber orientation after applying polarity correction to the IC readings. Based on their work, the output correction factors applied in the present work were calculated from $S_{clin}$ given in mm via Equation (2).^22^

(2) $$k_{Q_{clin},Q_{ref}}^{f_{clin},f_{ref}}=\;6.752\cdot S_{clin}^{-2.316}+0.999$$

For the measurement of OFs and lateral dose profiles, the reference point of the IC (located 2.4 mm from the chamber tip on the chamber axis) was positioned in 10 cm water depth. Depth positioning accuracy was assumed to be $\sigma_{z}$ = 0.5 mm. Lateral dose profiles were measured with a step size corresponding to 1% of the nominal side length, for example, 0.1 mm for the 1 × 1 cm^2^ field. A fixed position on the CAX was used for the OF measurements. The CAX position was determined during lateral profile measurements with the highest scan resolution, that is, 0.1 mm, in the 1 × 1 cm^2^ field. The lateral accuracy of positioning the IC on the CAX for all field sizes was thus 0.1 mm. For the determination of OFs, 200 monitor units (MU) were applied for all field sizes. Three irradiations per polarity and field size were performed. The electrometer readings were corrected for air density and the polarity effect. The applied polarity correction factor was determined via

(3) $$k_{pol}=\frac{M_{+U}+\left|M_{-U}\right|}{2\;M_{+U}}\;$$

where $M_{+U\;}$ and $M_{-U\;}$ define the electrometer readings for positive and negative voltage *U*, respectively.

The ratio of the electrometer readings of each field size versus the reference 10 × 10 cm^2^ field were further corrected by the correspondent small field output correction factor $k_{Q_{clin},Q_{ref}}^{f_{clin},f_{ref}}$ (Table 1).^2^, ^22^ This correction factor considers volume averaging and density effects in small fields.^22^ The corresponding relative standard uncertainty $u_{r}\left(k_{Q_{clin},Q_{ref}}^{f_{clin},f_{ref}}\right)$ was reported as 0.5% (1σ).^2^, ^22^ The influence of field size on the effect of incomplete saturation was found to be negligible for the field sizes investigated in the present work. Nevertheless, a relative standard uncertainty $u_{r}\left(k_{S}\right)$ *=* 0.02% was considered in the uncertainty budget. Uncertainties concerning the electrometer were estimated as 0.5% for random effects and 0.06% due to the limited resolution of the reported results as proposed in DIN6809‐8.^2^

**TABLE 1:** Values of the small field correction factor $k_{Q_{clin},Q_{ref}}^{f_{clin},f_{ref}}$ for the PinPoint 3D chamber.

Field size (cm^2^)	1 × 1	2 × 2	3 × 3	4 × 4
$k_{Q_{clin},Q_{ref}}^{f_{clin},f_{ref}}$	1.032 (5)	1.006 (5)	1.002 (5)	1.000 (5)

*Note*: Values obtained via Equation (2) for parallel orientation, SSD = 90 cm, depth in water = 10 cm. The number in parentheses corresponds to the reported uncertainty of 0.5% (1σ).^2^, ^21^

#### 2.2.2 Alanine pellets

**FIGURE 1:**
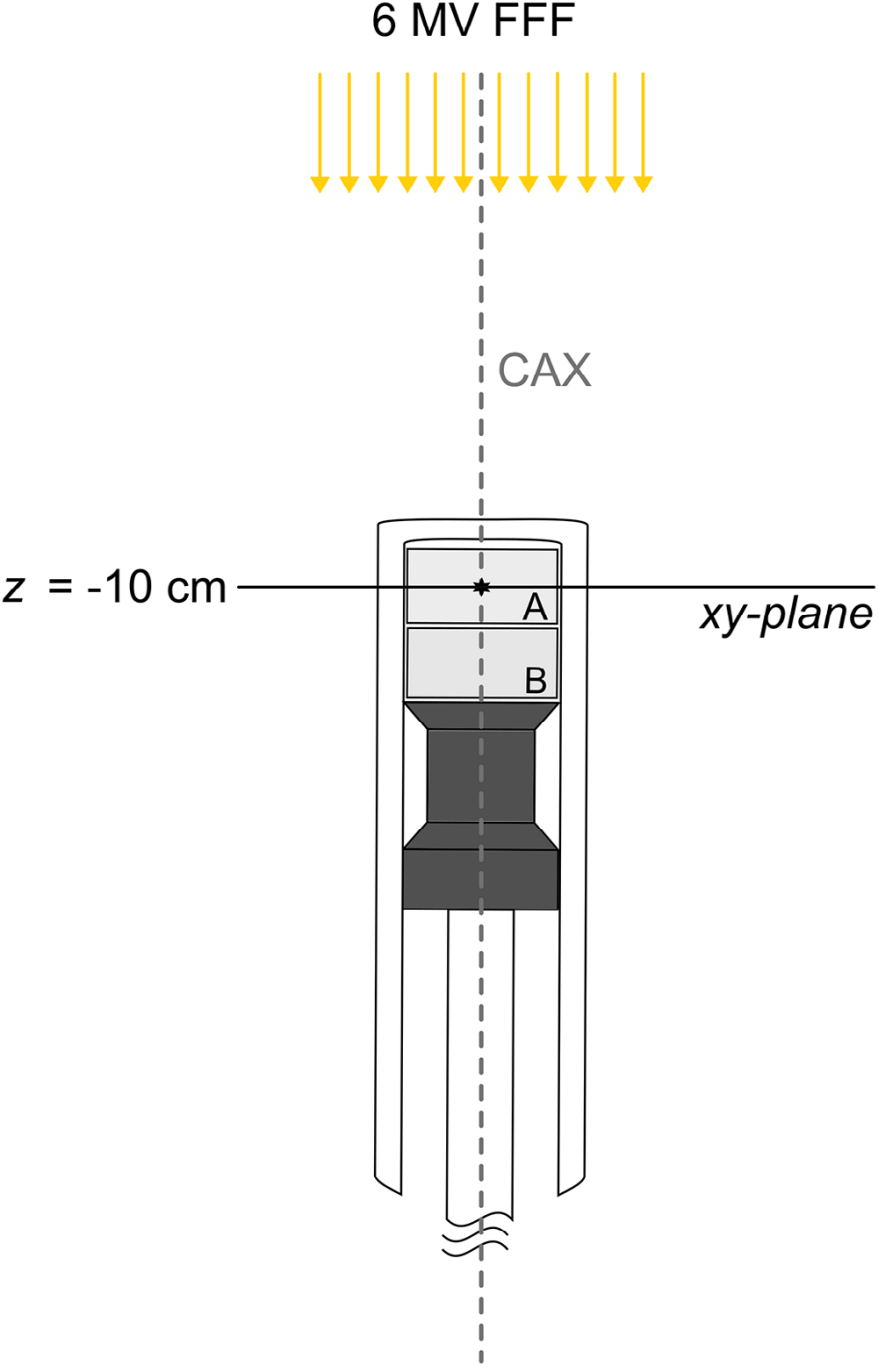
Schematic sketch of the alanine dosimeter design. Two alanine pellets (A) and (B) were placed into one cylindrical polyethylene dosimeter capsule. For irradiation, the center of the top pellet (A) was positioned on the CAX and in the measurement depth of 10 cm in water. Only pellet (A) was evaluated. Waterproof sealing was realized with a synthetic rubber plug (colored black). The plug is connected to a polystyrene stem for the purpose of better handling. CAX, central axis.

For EPR dosimetry, L‐alanine pellets with a diameter *d* of 4 mm, height *h* of 2.4 mm and mass of (35.87 ± 0.05) mg (1σ) were used. As in our previous work,^8^, ^15^, ^16^ pellets were stored in a controlled environment at room temperature and a relative humidity of 34% ± 2% for at least 8 weeks prior to first use. To enable irradiations inside the water tank phantom, the pellets were put into water‐tight cylindrical capsules made of low‐density polyethylene (Figure 1). In total, 15 capsules were prepared, each containing two ALA pellets but only the top pellet (A) was evaluated. The bottom pellet (B) served as a spacer for reducing possible fluence perturbation of the synthetic rubber plug used for sealing. The capsules were connected to a stem made of polystyrene for improved handling in the water tank. In the following, the unit of both elements (capsule and stem) are referred as ALA detector.

ALA irradiations were performed after IC measurements using the same detector holder of the water tank in the same lateral (xy‐plane) position. Thus, the capsules were positioned in parallel orientation to the beam as shown in Figure 1. Lateral positioning accuracy in relation to the CAX was inferior compared to the IC positioning, since the fixation of the ALA detector to the dosimeter holder introduced an additional uncertainty. A correction procedure that accounts for the underdosing resulting from lateral displacements is outlined in Section 4.

The geometric center of the top pellet was placed in the measurement depth of 10 cm in water. As for the IC, depth positioning accuracy was assumed to be $\sigma_{z}$= 0.5 mm. Three ALA detectors were irradiated per field size. 2650 MUs were applied per irradiation. For the 10 × 10 cm^2^ field size, the chosen number of 2650 MUs corresponded to an absorbed dose of 20.0 Gy in the top alanine pellet. The ALA readings were calibrated to the mean reading obtained from the three pellets irradiated in the 10 × 10 cm^2^ field.

EPR readout was performed within 1 week after irradiation using a MiniScope MS 5000 benchtop spectrometer (Magnettech by Freiberg Instruments, Freiberg, Germany) operating in X‐band. In the first step, dose evaluation was performed according to the procedure described in detail in our previous work^8^: Signal amplitudes were evaluated via spectral fitting with pre‐known base spectra. Resulting ALA signal intensities were corrected for individual pellet mass and for sensitivity fluctuations of the spectrometer via a manganese reference signal. The following uncertainties were considered. For the determination of pellet mass an absolute uncertainty of u(m) *=* 0.05 mg was assumed resulting in a relative standard uncertainty of $u_{r}\left(m\right)$ *=* 0.13%. Water temperature was monitored and was within 20.3°C and 21.2°C. Irradiation temperature was not corrected for but considered in the uncertainty budget. An absolute uncertainty of u(T) *=* 1 K was assumed for the dosimeter temperature during irradiation which leads to $u_{r}\left(T\right)\;$ *=* 0.16%. The relative uncertainty of the reproducibility of irradiation dose was $u_{r}\left(D_{irr}\right)$ *=* 0.2%. A calibration curve and an uncertainty model was applied to convert signal intensities to dose values $D_{ALA}$ and to predict relative dose uncertainties $u_{r}\left(D_{ALA}\right)$. The applied absolute dose values enabled relative dose uncertainties $u_{r}\left(D_{ALA}\right)$ below 1% for all field sizes.^8^ $D_{ALA}$ reflects the average dose absorbed by the ALA pellet and may differ from the point dose on the CAX which is relevant for OF measurements. Therefore, in a second step, corrections regarding volume averaging were applied (cf. Section 3) and further corrections resulting from positional uncertainties were taken into account (cf. Section 4).

#### 2.2.3 Radiochromic film

A single batch of gafchromic EBT3 film was used. When films were not in use, they were stored at room temperature in a light‐tight envelope. Film dosimetry was performed following the multichannel protocol presented by Lewis et al.^23^

Film scanning was performed with an Epson Perfection V750 Pro multicolor flatbed scanner (Seiko Epson Corporation, Suwa, Japan) at 72 dpi resolution and in 48‐bit red‐green‐blue (RGB) format. Image correction functions were disabled. Film scanning was conducted 24 h after irradiation and in portrait orientation only, that is, the long side of the original 20.3 × 25.4 cm^2^ film was parallel to the scan direction. Prior to scanning, the scanner was warmed up by three preview scans. A glass compression plate was used during scanning to avoid the influence of film curvature on the results as reported by Palmer et al.^24^ Film pieces were scanned with their center aligned on the central scanner axis to reduce the influence of lateral scan artifacts.^23^ The three RGB color channels were averaged and processing included a rescaling process as suggested by Lewis et al.^23^

### 2.3 Determination of the volume averaging correction factor $k_{vol}$ for ALA

**FIGURE 2:**
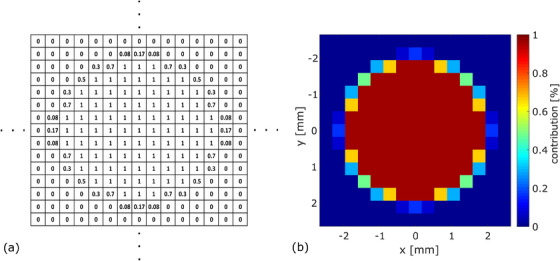
Numeric (a) and graphical (b) representation of normalized contribution matrix *W* given as percentages for 72 dpi scan resolution and a pellet diameter of d= 4 mm.

To account for volume averaging effects due to the finite size of the ALA pellets, a correction factor was determined using the 2D relative dose distribution obtained from film measurement in the xy‐plane (defined in Figure 1) at the same source‐to‐detector distance. The film dose values were first normalized to the dose value at the CAX. Afterwards, the average dose $D_{mean}\left(n\times \;n\right)$ within a circular area with diameter *d* *=* 4 mm (corresponding to the pellets cross section in the xy‐plane) was evaluated for each n×n cm^2^ field size. For this purpose, a weighing matrix *W* was defined for 72 dpi scan resolution reflecting the normalized contribution of each voxel to $D_{mean}\left(n\times \;n\right)$ (Figure 2). The center pixel of *W* coincided with the center pixel of the film dose matrix defining the CAX pixel. $D_{mean}\left(n\times \;n\right)$ was calculated by multiplication of *W* with the film dose matrix element by element and subsequent summation. The resulting field size dependent volume correction factor $k_{vol}\left(n\times \;n\right)$ was derived according to:

(4) $$k_{vol}\left(n\times n\right)=1/D_{mean}\left(n\times n\right)$$

Only 2D data in the xy‐plane was evaluated. The third z‐dimension corresponding to the pellet height was neglected as the pellets geometric center was positioned in the measurement depth of 10 cm and percentage depth dose curves (PDDs) could be assumed to fall off linearly within the range of the pellet height (*h* *=* 2.4 mm).

Uncertainties for the volume averaging correction factor $u_{r}\left(k_{vol}\left(n\times \;n\right)\right)$ were estimated for each n×n cm^2^ field size by re‐evaluating $k_{vol}\left(n\times \;n\right)$ when shifting the center of *W* to each of the 8 pixels adjacent to the CAX pixel. $u_{r}\left(k_{vol}\left(n\times \;n\right)\right)$ was defined as the standard deviation of the resulting nine $k_{vol}\left(n\times \;n\right)$ values for each field size.

### 2.4 Impact of positional uncertainties

Besides volume averaging, also incorrect positioning, that is, displacement form the CAX, affects the dose to the ALA pellets in small fields due to their lateral extent (diameter of 4 mm). The effect of incorrect positioning on the dose to the ALA pellet is expected to result in an underdosing. The present study accounts for this systematic underestimation of the true CAX dose by applying a correction factor $k_{pos}\left(n\times \;n\right)$ for ALA which is derived in the following.

If one assumes that lateral dosimeter displacements, that is, in *x*‐ and *y*‐ directions, are independent and normally distributed around the CAX (x=0,y=0), the probability distribution for a certain displacement vector $\vec{r}=\left(x,y\right)\;$ can be expressed as a bivariate normal distribution

(5) $$P\;\left(x,y\right)=\;C\cdot \exp\left(-\frac{x^{2}}{2\sigma_{x}}-\frac{y^{2}}{2\sigma_{y}}\right)$$

with a normalization factor *C*. If one further assumes that the standard deviation in *x*‐ and *y*‐ directions are equal $\sigma_{x}=\sigma_{y}\;=\sigma_{x,y}\;$, Equation (5) is reduced to

(6) $$P\;\left(x,y\right)=\;C\cdot \exp\left(-\frac{x^{2}+y^{2}}{2\sigma_{x,y}}\right).$$

The probability for a displacement distance $r\;=\sqrt{x^{2}+y^{2}}\;$, that is, the distance from the CAX, is then given by

(7) $$P\;\left(r\right)=C^{\prime }\;\cdot r\cdot \exp\left(-\frac{r^{2}}{2\sigma_{x,y}}\right)$$

with a normalization factor $C^{\prime }$. The first derivative of Equation (7) reveals that the maximum of P(r) is located at $r\;=\sigma_{x,y}\;$.

In the current work, correction factors $k_{pos,\;r\;mm}\left(n\times \;n\right)$ for different displacement distances *r* were derived from 2D film dose measurements similar to $k_{vol}\left(n\times \;n\right)$ (cf. Section 3). The weighing matrix *W* was either centered to the CAX pixel of the film dose matrix resulting in $D_{mean,\;0\;mm}$ or shifted by *r* mm from the field center in +*x*, ‐*x*, +*y* and ‐*y* direction resulting in four mean dose values $D_{mean,\;(x,y)}$ ($D_{mean,\;(+r\;mm,\;0)},D_{mean,\;(-r\;mm,\;0)}$, $D_{mean,\;(0,\;+r\;mm)}\;$and $D_{mean,\;(0,-r\;mm)}$). The average and standard deviation of the latter four values were defined as $D_{mean,\;r\;mm}$ and $D_{SD,\;r\;mm}$, respectively.

$k_{pos,\;r\;mm}$ was defined as the ratio

(8) $$k_{pos,\;r\;mm}=D_{mean,\;0\;mm}/D_{mean,\;r\;mm}.$$

The corresponding relative uncertainty was estimated via

(9) $$u_{r}\left(k_{pos,\;r\;mm}\right)=D_{SD,\;r\;mm}/D_{mean,\;r\;mm}$$

The outlined procedure was performed for four displacement values *r* (0.35, 0.71, 1.06 and 1.41 mm) corresponding to 1, 2, 3 and 4 pixel shifts for each investigated field size in order to investigate the dependence of $k_{pos}$ on $\sigma_{x,y}$.

For the IC measurements, a lateral positional accuracy of $\sigma_{x,y}$ = 0.1 mm was assumed. The resulting dose uncertainty for each field size was estimated by evaluating the water tank profile (inline and crossline) measurements ± 0.1 mm from the zero (CAX) position. The average deviation of the resulting dose values form the CAX value was taken as uncertainty $u_{r}\left(pos\right)$.

Evaluation of the PDD slope in the measurement depth ± 0.5 mm led to the relative positioning uncertainty $u_{r}\left(PDD\right)$ applied to both detector types.

### 2.5 Beam model data

OFs were also extracted from Halcyon's pre‐configured BM (Eclipse Version 15.6.06). The values are normalized to the 10 × 10 cm^2^ field size and are valid for the irradiation setup described in methods Section 1. As the BM does not include an OF value for the field size of 3 × 3 cm^2^, an analytical function introduced by Sauer and Wilbert^25^ (Equation (10)) was fitted to the available OF data (including values for 6 × 6 cm^2^ and 8 × 8 cm^2^) to obtain an appropriate intermediate value at a square field side length *s* of 3 cm.

(10) $$OF\;\left(s\right)=P_{\infty }\;\frac{S^{n}}{l^{n}+S^{n}}+S_{\infty }\left(1-e^{-bS}\right)$$

$P_{\infty }$ can be considered as the maximum primary dose component; $S_{\infty }$ is the maximum scatter component; *l*, *n* and *b* are fit parameters.

## 3 RESULTS

### 3.1 Volume averaging correction factor $k_{vol}$ for ALA

**FIGURE 3:**
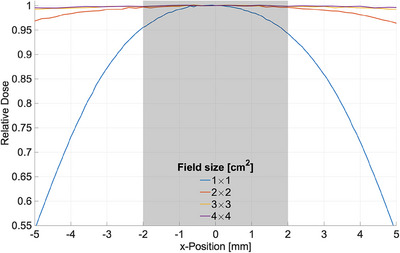
CAX normalized dose profiles obtained with the PinPoint 3D ionization chamber in relation to the lateral dimension of an alanine pellet of diameter *d* = 4 mm (grey area) illustrating the volume averaging effect. In case of a field size of 1 × 1 cm^2^ the lateral dose fall‐off affects the measured average dose within the pellet. CAX, central axis.

Figure 3 shows the dimensions of an alanine pellet in comparison to CAX normalized lateral dose profiles obtained with the IC in the water tank. With their diameter of 4 mm the pellets were expected to show substantial volume averaging effects in the 1 × 1 cm^2^ field. Whereas in the 2 × 2 cm^2^ field the profiles are quite flat across the dimensions of a centered pellet.

**FIGURE 4:**
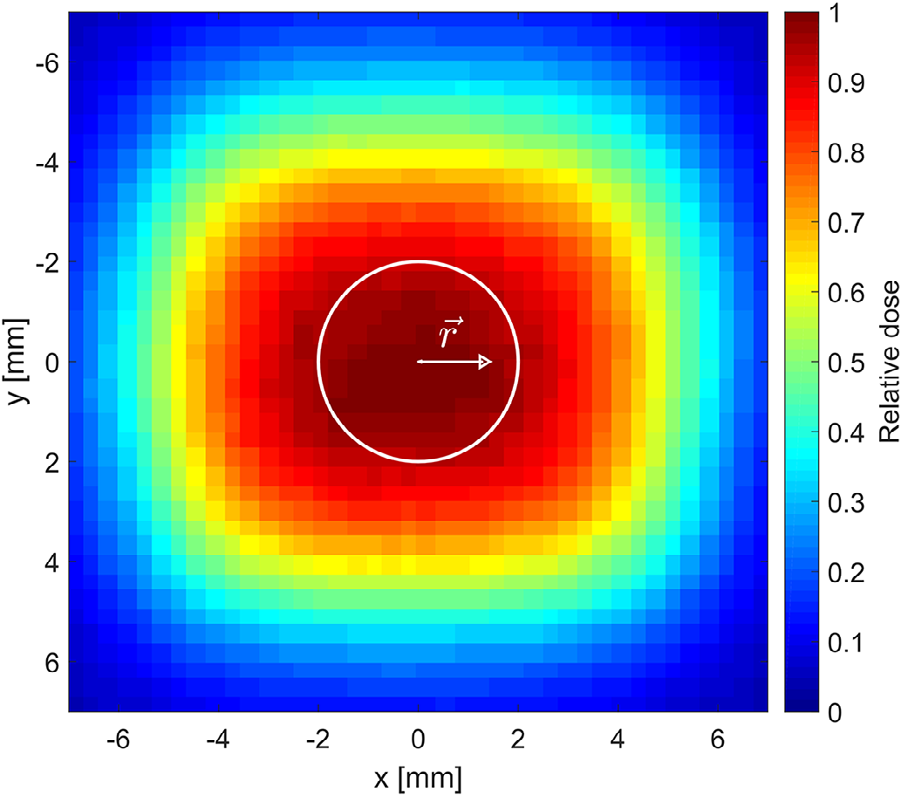
Example of a CAX normalized 2D dose distribution measured with gafchromic EBT3 film for the 1 × 1 cm^2^ nominal field size. The white circle visualizes the cross section (*d* *=* 4 mm) of an alanine pellet. For the determination of $k_{pos,r\;mm}$film dose values within the cross section were averaged after shifting the circle by $|\vec{r}|$ in ±*x*, ±*y* as described in the text. CAX, central axis.

For a detailed analysis, 2D film dose profiles were evaluated in the circular dosimeter cross section (Figure 4) to obtain an average measured dose $D_{mean}\left(n\times \;n\right)$ relative to the maximum dose at field center. The resulting values of $D_{mean}\left(n\times \;n\right)$ are shown in Table 2 for each field size together with the derived volume averaging correction factors $k_{vol}\left(n\times \;n\right)$ (Equation 4). The analysis shows that volume averaging correction starts becoming relevant for field sizes below 2 × 2 cm^2^, which corresponds qualitatively to the illustration in Figure 3. Neglecting volume averaging effects would lead to an underestimation of the CAX dose by about 3% in the 1 × 1 cm^2^ field. It should be noted that the resulting volume correction factors are specific to the beam geometry in the measurement setup and the pellet dimensions.

**TABLE 2:** Relative dose $D_{mean}$ and correction factor $k_{vol}$ for each nominal field size.

Field size (cm^2^)	1 × 1	2 × 2	3 × 3	4 × 4	10 × 10
$D_{mean}$(%)	97.0 (2)	99.9 (1)	100.0 (1)	100.0 (1)	100.0 (1)
$k_{vol}$	1.030(2)	1.001 (1)	1.000 (1)	1.000 (1)	1.000 (1)

*Note*: $D_{mean}$ was obtained by averaging 2D film data across circular area reflecting the pellet cross section as described in the text. Volume averaging correction factors were determined via Equation (4). The number in parentheses is the estimated absolute uncertainty referred to the corresponding last digits of the reported result.

The relative uncertainties of the volume averaging correction factors were determined to be $u_{r}\left(k_{vol}\left(1\times \;1\right)\right)$ *=* 0.2% and $u_{r}\left(k_{vol}\left(2\times \;2\right)\right)$, $u_{r}\left(k_{vol}\left(3\times \;3\right)\right)$, $u_{r}\left(k_{vol}\left(4\times \;4\right)\right)$ *=* 0.1%. No volume averaging correction was applied in the 10 × 10 cm^2^ field and there was no additional uncertainty considered.

### 3.2 Correction factor $k_{pos}$ for ALA

Table 3 shows the resulting correction factors $k_{pos}$ for each field size and for four different displacement distances *r* (0.35, 0.71, 1.06 and 1.41 mm), that is, assuming different positional uncertainties $\sigma_{x,y}$ accordingly (cf. methods Section 4). By comparing the magnitude of the proposed correction factors with their corresponding uncertainties (given in parentheses in Table 3) it can be noted that relevant and reliable correction factors $k_{pos}$ deviating from unity could only be obtained for the 1 × 1 cm^2^ field size. $k_{pos}$ is increasing with increasing positional uncertainty $\sigma_{x,y}$ for the 1 × 1 cm^2^ field. The obtained results predict an underestimation of about 0.2%, 0.8%, 1.8% and 3.3% when the ALA pellet is displaced by 0.35, 0.71, 1.06 and 1.41 mm from the CAX in the 1 × 1 cm^2^ field, respectively. For the other field sizes $k_{pos}$ was negligible.

**TABLE 3:** Correction factor $k_{pos}$ for each nominal field size.

Field size (cm^2^)	1 × 1	2 × 2	3 × 3	4 × 4	10 × 10
$k_{pos,\;0.35\;mm}$	1.002 (1)	1.000 (1)	1.000 (1)	1.000 (1)	1.000 (1)
$k_{pos,\;0.71\;mm}$	1.008 (3)	1.000 (1)	1.000 (1)	1.000 (1)	1.000 (1)
$k_{pos,\;1.06\;mm}$	1.018 (6)	1.000 (1)	1.001 (2)	1.000 (1)	1.000 (1)
$k_{pos,\;1.41\;mm}$	1.033(11)	1.001(1)	1.001(3)	1.001(1)	0.999 (2)

*Note*: $k_{pos}$ correction factors for different displacement distances *r* (0.35, 0.71, 1.06 and 1.41 mm) from the CAX. The corresponding standard uncertainties are given in parentheses.

### 3.3 Individual and mean ALA results before applying $k_{vol}$ and $k_{pos}$

Table 4 shows the EPR dosimetry results (absolute dose values $D_{ALA}$ and OFs) obtained by measuring and evaluating three irradiated ALA dosimeters per field size. The combined absolute uncertainties (1σ) originating from the EPR dosimetry procedure and error propagation are given in parentheses. Due to normalization of the OF at 10 × 10 cm^2^, the uncertainty u(10×10) is included in these values. Combined relative standard uncertainties for the OFs resulted in $u_{c}\left(1\times 1\right)$ *=* 1.24%, $u_{c}\left(2\times 2\right)\;$ *=* 1.12%, $u_{c}\left(3\times 3\right)$ *=* 1.09%, $u_{c}\left(4\times 4\right)$ *=* 1.07%. For each field size, the individual results of the three ALA dosimeter readouts are in good agreement with a maximum difference below 1.8%. $u_{r}\left(PDD\right)$ was found to be 0.3%.

**TABLE 4:** Absolute dose values and output factors determined with L‐alanine before applying $k_{vol}$ and $k_{pos}$ for each nominal field size.

Field size (cm^2^)	1 × 1	2 × 2	3 × 3	4 × 4	10 × 10
$D_{ALA}$ #1 (Gy)	13.44 (14)	16.06 (14)	16.87 (14)	17.50 (14)	19.97 (14)
$D_{ALA}$ #2 (Gy)	13.54 (14)	16.24 (14)	17.06 (14)	17.69 (14)	19.89 (14)
$D_{ALA}$ #3 (Gy)	13.54 (14)	16.35 (14)	16.86 (14)	17.74 (14)	20.15 (14)
Mean $D_{ALA}$	13.51 (14)	16.22 (14)	16.93 (14)	17.65 (14)	20.00 (14)
OF #1	0.672 (8)	0.803 (9)	0.844 (9)	0.875 (10)	0.999
OF #2	0.677 (8)	0.812 (9)	0.853 (9)	0.885 (10)	0.994
OF #3	0.677 (8)	0.817 (9)	0.843 (9)	0.887 (10)	1.007
Mean OF	0.675 (8)	0.811 (9)	0.847 (9)	0.882 (10)	1.000

*Note*: Measured absolute dose values $D_{ALA}$ (averaged across pellet volume) for the individual ALA pellets denoted as #1, #2, and #3. The number in parentheses is the absolute dose uncertainty $u(D_{ALA})$ (1σ) originating from the applied EPR dosimetry protocol. Output factors normalized to the mean ALA dose for a nominal field size of 10 × 10 cm^2^. The number in parentheses is the resulting combined absolute uncertainty $u_{c}$ (1σ) referred to the corresponding last digits of the reported result.

Abbreviation: ALA, L‐alanine; OFs, output factors.

For ALA, a summary of the considered uncertainty contributions is presented in Table 5.

**TABLE 5:** Uncertainty budget for ALA L‐alanine.

Field size (cm^2^)	1 × 1	2 × 2	3 × 3	4 × 4	10 × 10
$u_{r}(D_{ALA}$) (%)	1.01	0.86	0.83	0.80	0.72
$u_{r}(k_{vol}$) (%)	0.20	0.10	0.10	0.10	–
$u_{r}(k_{pos,\;1.06\;\mathrm{mm}}$) (%)	0.60	0.10	0.10	0.10	–
$u_{r}(PDD$) (%)	0.30	0.30	0.30	0.30	0.30
$u_{c}(OF$) (%) uncorrected	1.24	1.12	1.09	1.07	–
$u_{c}(OF$) (%) corrected ($k_{vol}$)	1.33	1.20	1.18	1.16	–
$u_{c}(OF$) (%) corrected ($k_{vol},k_{pos,\;1.06\;\mathrm{mm}}$)	1.46	1.21	1.18	1.16	–

*Note*: Relative standard uncertainty (1σ) components $u_{r}$ considered in the determination of OFs with ALA. Resulting combined relative uncertainties were obtained via Gauss’ law of uncertainty propagation.

Abbreviation: ALA, L‐alanine; OFs, output factors.

### 3.4 IC results and uncertainties

Following Equation (1), the OF results obtained after applying $k_{Q_{clin},Q_{ref}}^{f_{clin},f_{ref}}$ (Table 1) to the IC readings (corrected for influence quantities) are shown in Table 6 together with the estimated combined absolute uncertainties (1σ) given in parentheses. All uncertainty contributions mentioned in methods Section 2.1 as well as uncertainties accounting for positional inaccuracies ($u_{r}\left(pos\right)$ and $u_{r}\left(PDD\right)$) are listed in Table 7 and were taken into account. Relative uncertainties accounting for 0.1 mm lateral positioning inaccuracy were found to be small ($u_{r}\left(pos\right)$ *<* 0.1% for all field sizes). As for ALA, $u_{r}\left(PDD\right)$ for IC was estimated to be 0.3%. Resulting combined relative standard uncertainties (1σ) were $u_{c}(OF$) *=* 0.97% for all field sizes.

**TABLE 6:** Comparison of corrected measured output factors and beam model data.

Field size (cm^2^)	1 × 1	2 × 2	3 × 3	4 × 4	10 × 10
Output factors					
BM	0.697	0.804	*0.848*	0.880	1.000
ALA ($k_{vol}$)	0.696 (9)	0.812 (10)	0.847 (10)	0.882 (10)	1.000
ALA ($k_{vol}$,$k_{pos,1.06\;mm}$)	0.708 (10)	0.812 (10)	0.847 (10)	0.882 (10)	1.000
IC	0.691 (7)	0.812 (8)	0.850 (8)	0.883 (9)	1.000
Deviations (%)					
ALA ($k_{vol}$)/IC	+0.7	0.0	–0.4	–0.1	
ALA ($k_{vol}$)/BM	–0.1	+1.0	–0.1	+0.2	
ALA ($k_{vol}$,$k_{pos,1.06\;mm}$)/IC	+2.5	0.0	–0.4	–0.1	
ALA ($k_{vol}$,$k_{pos,1.06\;mm}$)/BM	+1.6	+1.0	–0.1	+0.2	
IC/BM	–0.9	+1.0	+0.2	+0.3	

*Note*: The beam model (BM) value for 3 × 3 cm^2^ shown in italic font is resulting from the fitted analytical function (Equation 10). For alanine, values include either only the volume averaging correction $k_{vol}$ (ALA ($k_{vol}$)), or both $k_{vol}$ and $k_{pos,1.06\;mm}$ (ALA ($k_{vol}$,$k_{pos,1.06\;mm}$)). For the PinPoint 3D (IC), values include the small field correction $k_{Q_{clin},Q_{ref}}^{f_{clin},f_{ref}}$. The numbers in parentheses show the combined absolute uncertainties (1σ) referred to the corresponding last digits of the reported result. The stated uncertainties for ALA include the contributions $u(D_{ALA})$, u(PDD), $u(k_{vol})$. The uncertainty of ALA ($k_{vol}$,$k_{pos,1.06\;mm}$) for the 1 × 1 cm^2^ field size includes $u(k_{pos,1.06\;mm})$ additionally.

Abbreviations: BM, beam model; ALA, L‐alanine; IC, ionization chamber.

**TABLE 7:** Uncertainty budget for ionization chamber measurements.

Field size (cm^2^)	1 × 1	2 × 2	3 × 3	4 × 4	10 × 10
$u_{r}(k_{S}$) (%)	0.02	0.02	0.02	0.02	0.02
$u_{r}(k_{Q_{clin},Q_{ref}}^{f_{clin},f_{ref}}$) (%)	0.50	0.50	0.50	0.50	–
$u_{r}\left(pos\right)$ (%)	0.05	0.05	0.01	0.01	0.01
$u_{r}(PDD$) (%)	0.30	0.30	0.30	0.30	0.30
Electrometer:					
Random effects (%)	0.50	0.50	0.50	0.50	0.50
Limited resolution (%)	0.06	0.06	0.06	0.06	0.06
$u_{c}(OF$) (%) corrected ($k_{Q_{clin},Q_{ref}}^{f_{clin},f_{ref}}$)	0.97	0.97	0.97	0.97	–

*Note*: Relative standard uncertainty (1σ) components $u_{r}$ considered in the determination of OFs with the PinPoint 3D 31022 chamber. Resulting combined relative uncertainties for the corrected OFs were obtained via Gauss’ law of uncertainty propagation.

Abbreviation: OFs, output factors.

### 3.5 Modeling OF data

**FIGURE 5:**
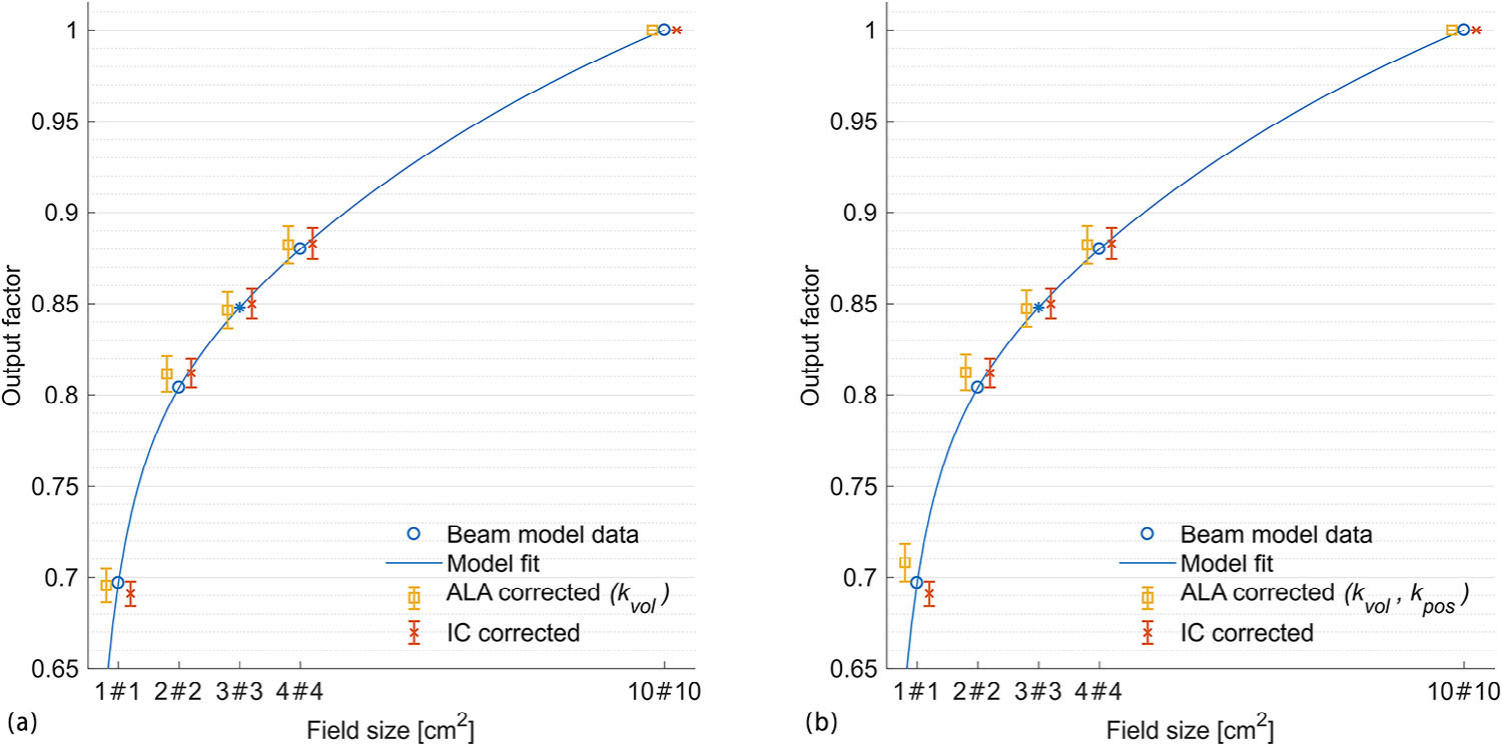
Measured output factors in comparison to the corresponding manufacturer provided beam model data. Measured values are shifted to the left/right for better readability. The output factor of the beam model for the 3 × 3 cm^2^ (shown as asterisk) is derived from a model fit as described in the text. Error bars indicate the resulting combined uncertainty (1σ). (a) Corrected results including the volume averaging correction $k_{vol}$ for alanine and $k_{Q_{clin},Q_{ref}}^{f_{clin},f_{ref}}$ for the PinPoint 3D IC. (b) Additional application of correction factor $k_{pos,1.06\;mm}$ to the ALA result for 1 × 1 cm^2^ field size. ALA, L‐alanine.

The curve resulting from a least‐squares fit of the proposed analytical function (Equation 10) to the BM OF data is shown in Figure 5. The optimal curve parameters were:

$P_{\infty }$ = 0.754, $S_{\infty }\;$= 0.374, *l* = 0.421, *n* = 2.242 and *b* = 0.108.

### 3.6 Comparison of output factors and the influence of $k_{pos}$

OFs determined by ALA and IC measurements as well as those incorporated in the BM are illustrated in Figure 5. Figure 5a shows corrected results, that is, after applying $k_{vol}$ for ALA (Table 2) and $k_{Q_{clin},Q_{ref}}^{f_{clin},f_{ref}}$ for IC. Vertical error bars indicate the resulting combined uncertainties (1σ). Relative combined uncertainties were lower than 1.4% for both dosimeter types (ALA and IC) and for all investigated field sizes. Within these uncertainties, corrected measurement results agree well with the BM in the 1 × 1, 2 × 2, 3 × 3 and 4 × 4 cm^2^ fields. The largest deviation is found for the 2 × 2 cm^2^ field. Here, the BM lies 1.0% below the measured values. The maximum differences between ALA and IC results was 0.7% occurring for the smallest field size (1 × 1 cm^2^).

In Figure 5b, a positional uncertainty of $\sigma_{x,y}\;$= 1.06 mm is exemplarily assumed and the ALA result for the 1 × 1 cm^2^ field size is corrected by the corresponding correction factor $k_{pos}$. Compared to Figure 5a, the resulting OF for the 1 × 1 cm^2^ field is thus increased. The relative difference to the BM and the IC result amounts to 1.6% and 2.5%, respectively (Table 6). In this case, the (1σ) uncertainty range of the ALA result indicated by the error bars does not include the values of the BM and the IC result. For the other field sizes, the influence of positional uncertainties was negligible (cf. Table 3).

## 4 DISCUSSION

The focus of the present work was to provide procedures how to quantify the volume averaging effect as well as how to elucidate the effect of positional uncertainties for OF measurements with ALA in small fields. With the ALA pellets used in this work, both effects need to be taken into account for accurate OF determinations, especially for field sizes < 2 × 2 cm^2^.

A practical EPR dosimetry procedure tailored for routine use in radiotherapy was applied using commercially available ALA pellets with a diameter of 4 mm. The finite pellet size leads to a non‐negligible underestimation of dose by 3.0% in the 1 × 1 cm^2^ field (Table 2) due to the volume averaging effect requiring application of a correction factor $k_{vol}$. The correction factor determined in this work is slightly higher compared to volume averaging correction factors reported in the literature (about 1.01 for a 1.2 × 1.2 cm^2^ field and about 1.02 for a 0.9 × 0.9 cm^2^ field),^3^ where a comparable method for determining $k_{vol}$ was applied and even larger pellets with a diameter of 5 mm were used. The discrepancy may be attributed to differences in the lateral dose distribution due to, for example, different beam shaping components (collimators, jaws) or beam characteristics (flattening filter vs. FFF).

For field sizes above 2 × 2 cm^2^ the volume averaging effect is already below 0.1%.

Regarding positional accuracy, our analysis shows that displacements from the CAX in the order of 0.7–1.4 mm lead to a dose underestimation of 0.8%–3.3%. This finding emphasizes the importance of careful positioning and setup verification when using commercial ALA pellets with a size of several mm in small fields (< 2 × 2 cm^2^). In the present work, a correction factor $k_{pos}$ was determined in order to account for lateral positional uncertainties. Based on our experience lateral positional uncertainties better than $\sigma_{x,y}\;$= 1.0 mm are hardly achievable for the used ALA dosimeters even though the same dosimeter holder position was used as for the IC measurements. A corresponding correction factor $k_{pos,\;1.06\;mm}\;$= 1.018 for the 1 × 1 cm^2^ field size could be determined from 2D film dose measurements.

Vertical positional uncertainties were incorporated in the uncertainty budget as $u_{r}\left(PDD\right)$.

By applying solely volume averaging correction factors $k_{vol}$ the ALA results agree within uncertainties with the independent IC measurement results. The latter are obtained by applying a well‐established small field dosimetry protocol.^1^ Moreover, the OFs extracted from the BM data are within the combined uncertainties (1σ) of the corrected ALA ($k_{vol}$) results (cf. Figure 5a and Table 6). The Halcyon's BM is pre‐configured and cannot be adjusted by the user. Instead, the machine is tuned to this model during commissioning. Small field OFs of this BM down to 1 × 1 cm^2^ have already been validated by measurements within 2%.^18^ The IC results obtained in the present work agree well with the BM OF data shown in Table 6 and Figure 5. The maximum difference of 1.0% was observed for the 2 × 2 cm^2^ field size.

When correcting for positional uncertainties by applying a reasonable correction factor $k_{pos,\;1.06\;mm}$, the OF value obtained via ALA dosimetry for the 1 × 1 cm^2^ field size exceeds the value from IC measurement and the BM value by 2.5% and 1.6%, respectively. This finding may indicate the existence of a non‐negligible density effect that was already postulated by Cronholm et al.^14^ They performed a Monte Carlo (MC) study of the ALA response in small fields of a 6 MV photon beam and claimed a non‐negligible effect of the non‐water composition of ALA detectors in situations where CPE is lost, for example, when the field size is smaller than the range of the secondary electrons. The volume averaged dose to alanine was compared to the volume averaged dose to water for the same detector size thereby excluding volume averaging effects from their analysis. According to the results of their study, the response of alanine detectors with a diameter of 5 mm increases relative to water by about 1.5% when the field size is decreased to 1 × 1 cm^2^. This result translates into an increase of measured small field OFs if the density effect is not considered—as observed in the current work. In summary, correcting for positional uncertainties leads to increased OF results. Correcting for the density effect would lead to lowered OF results. Both corrections may become relevant in small fields < 2 × 2 cm^2^.

In the present study, volume averaging and positional uncertainties were taken into account during evaluation. The density effect, however, was not considered due to the unavailability of appropriate data for the used ALA pellets.

Other researchers solely applied corrections for volume averaging to their ALA results and used the ALA response as reference.^3^, ^12^ Based on the findings of the present work, this procedure may, but need not, be sufficient. Depending on the magnitudes of positional uncertainties and of the density effect both additional corrections may or may not compensate each other.

It is therefore crucial to investigate lateral positional uncertainties and their impact on the dose to the ALA dosimeters and, at the same time, to evaluate and consider a possible density effect. In our work, the estimated effect of positional uncertainties (1.8% for $\sigma_{x,y}\;$= 1.06 mm) as well as the estimated combined uncertainty for ALA (*k_vol,_k_pos_*) in the 1 × 1 cm^2^ field (about 1.5%) is in the same order as the density effect claimed by Cronholm et al. Further experimental studies are needed to prove the existence and relevance of the density effect in small fields (< 2 × 2 cm^2^).

In the present work we adapted an established EPR dosimetry procedure for measuring OFs in small fields with millimeter‐sized ALA dosimeters by taking additional corrections and uncertainties into account. A different approach is to reduce the pellet dimensions in order to reduce volume averaging and positioning issues. However, this approach leads, in turn, to increased EPR readout uncertainties $u_{r}(D_{ALA}$) due to the decreased pellet mass. Some studies investigated improved sensitivities for so called miniALA dosimeters by increasing the magnetic field strength during readout and, at the same time, operating at an increased microwave frequency (K‐band).^13^, ^26^, ^27^, ^28^ Under the current state of the art, this approach requires larger spectrometers. Moreover, miniALA are not yet commercially available.

## 5 CONCLUSION

Output factors measured with commercial alanine pellets are in good agreement with IC measurements and with the corresponding BM data. The alanine dosimetry procedure could be adapted for the determination of OFs in small fields by introducing volume averaging correction factors that were derived from 2D film dosimetry measurements. The adapted procedure is still precise (relative combined standard uncertainty *≤* 1.5%) for output factor determination in small fields down to 1 × 1 cm^2^ field size. Besides corrections for volume averaging, corrections for positional uncertainties may become relevant (effect > 1%) for field sizes smaller than 2 × 2 cm^2^ when alanine dosimeters with a diameter of 4 mm are used. The experimental results of the current work may indicate the existence of a non‐negligible density effect for field sizes smaller than 2 × 2 cm^2^. Further precise experimental studies that consider the effects of volume averaging and positional uncertainties are needed to underpin the existence and relevance of the density effect in small fields (< 2 × 2 cm^2^).

## AUTHOR CONTRIBUTIONS

Sebastian Höfel and Pauline Liebig performed the measurements, analyzed and visualized the data and wrote the manuscript. Michael K. Fix, Malte Drescher and Felix Zwicker supervised the work, revised the manuscript and provided substantial contribution to the aim, design and methodological approach of the presented work. All authors have given their consent for publication and approved the final version of the manuscript.

## CONFLICT OF INTEREST STATEMENT

The authors declare no conflicts of interest.
